# Editorial: Challenge and resilience: primary care in a COVID-19 world

**DOI:** 10.1017/S146342362100075X

**Published:** 2021-11-25

**Authors:** Felicity Goodyear-Smith

**Affiliations:** Department of General Practice & Primary Health Care, University of Auckland, PB 92019 Auckland, 1142, New Zealand. E-mail: f.goodyear-smith@auckland.ac.nz

Primary care is relationship-based care. Connection, communication and collective decision-making with our patients are key components. COVID-19 has disrupted the primary care landscape in many ways, and both clinicians and patients have experienced high levels of stress. The initial lockdowns in many countries meant that suddenly clinicians were unable to maintain the healing relationships with their patients. Primary care clinicians have reported increasing levels of mental exhaustion and burnout as the pandemic has progressed, in part due to their inability to provide the care their patients need.

When practices had to close their physical doors, income plummeted for many, and some primary care clinicians lost their jobs. The loss of practice income was compounded by additional costs such as financing personal protective equipment, requirement to adopt new information technologies, and reconfiguring their premises to triage possible COVID-19 cases and provide parallel segregated services for ‘red’ and ‘green’ streams. Hospitals stopped accepting referrals for non-COVID-19 patients, and urgent investigations and treatments were deferred.

Practices raced to connect with their patients remotely through telehealth, using phone, video, email, patient portals and other technologies. Innovative apps allowed patients to pass photos to and fro. However, some patients lacked access to mobile phones and the internet, and this digital divide exacerbated health care inequities (Watts, 2020).

Once countries’ borders were secured, primary health care (PHC) approaches could mitigate virus spread through public education to reduce person-to-person contact (using physical distancing measures, facial coverings and lockdown procedures), triage of cases, and COVID-19 testing, contact-tracing and surveillance. PHC comprises both population-level public health and individual-based primary care, as well as community-based social services (Muldoon et al., 2006). An international study found that in most countries, public health and primary care were insufficiently integrated for an effective epidemic response (Goodyear-Smith et al., 2021). For example, a coordinated approach to COVID-19 swabbing and contact-tracing of positive cases was often lacking.

The pandemic has impacted negatively on patients in a myriad of ways. This includes delays in accessing investigations or medical care, separation from loved ones, loss of jobs, interruption with education, and ongoing stress and mental health issues. Patients or their family members may have contracted COVID-19; friends or family may have died from the infection. People with long-term conditions such as diabetes and chronic cardiovascular, respiratory and renal disease have worse outcomes and increased mortality from COVID-19. Those infected may need intensive hospital services as well as community-based primary care support. At some times and places both of these have been unavailable, as services have become overwhelmed. A further consequence of lockdown and move to remote access consultations has been disruption of non-COVID-19 primary care for these patients. Further, as COVID-19 vaccination programmes progress, there have been disproportionately high rates of vaccine hesitancy in the more vulnerable ethnic minority and socio-economically deprived communities (Razai et al., 2021).

An additional burden on primary care is managing long COVID-19. About 90% of patients who test positive for SARS-CoV-2 virus recover within 3 weeks, but a small percentage remain unwell for weeks or months (Greenhalgh et al., 2020). Greenhalgh et al. define symptoms extending beyond 3 weeks as post-acute and beyond 12 weeks as chronic COVID-19. Symptoms can be wide-ranging, often multi-system, but fatigue and breathlessness often predominate. Most of these patients will be managed in primary care. Many will slowly recover with rest, psychosocial support, treatment of symptoms and graduated increase in physical activity. Breathless patients may be monitored using home pulse oximetry, but persistent or progressive symptoms at 3 months warrant specialist referral.

While the pandemic has created huge challenges for primary care, in many countries, positive impacts from the pandemic response also have been recognised. Our discipline is used to dealing with complexity and uncertainty, and in many areas primary care has demonstrated great resilience when finding solutions to the stressors COVID-19 has presented.

The pandemic response has enhanced team work, with task-shifting and clinicians working at the top of their scope. Collegial and supportive relationships between primary care providers have strengthened. In some countries, additional workforce has been mobilised, calling upon medical or nursing students, retired health professionals, volunteers or those in the non-government or private sectors (European Observatory on Health Systems and Policies *et al*., 2020).

The use of telehealth to connect with patients has brought many benefits including improved access for some patients. Digital tools help to support and manage patients remotely. The ability to prescribe, request investigations and refer electronically is resulting in improved coordination between primary and secondary care, and with community-based providers. Patients with long-term conditions can be supported through home-monitoring for biometrics such as weight, blood pressure and blood glucose, increasing both their access to care and their ability to self-manage.

To address growing health inequities such as food insecurity and poor housing, primary care is strengthening existing relationships and forging new partnerships with a broad range of services such as community pharmacies, mental health support, food banks and other social support agencies.

In many settings, population-based approaches to care have been strengthened with enhanced cooperation between public health and primary care. This applies both to COVID-19 identification and monitoring, and also now to the mass delivery of vaccinations. Primary care can provide outreach to vulnerable patients in their community, and their relationship with patients is a key to addressing vaccine hesitancy.

In many countries, cases are dropping steeply as the vaccine is rolled out. However, the world has changed. COVID-19 will remain endemic, and we need to stay vigilant. We have learnt that strong PHC, with well-integrated public health and primary care, will enable us to address the ongoing effects of this virus and have surveillance systems, preparedness plans and infrastructure in place to deal with another deadly pandemic in the future. Hopefully, the painful lessons have been learnt, and primary care meets the challenge and emerges as a key player in fighting infection and keeping our populations healthy.
